# The Impact of Surface Ligands and Synthesis Method on the Toxicity of Glutathione-Coated Gold Nanoparticles

**DOI:** 10.3390/nano4020355

**Published:** 2014-05-12

**Authors:** Bryan Harper, Federico Sinche, Rosina Ho Wu, Meenambika Gowrishankar, Grant Marquart, Marilyn Mackiewicz, Stacey L. Harper

**Affiliations:** 1 Department of Environmental and Molecular Toxicology, Oregon State University, 1007 ALS Building, Corvallis, OR 97331, USA; E-Mails: bryan.harper@oregonstate.edu (B.H.); fsinche@g.clemson.edu (F.S.); 2 Department of Chemistry, Portland State University, 1719 SW 10th Ave., Portland, OR 97201, USA; E-Mails: rosinah91@hotmail.com (R.H.W.); meenambika@gmail.com (M.G.); gwm@pdx.edu (G.M.); mackiewi@pdx.edu (M.M.)

**Keywords:** glutathione, gold, nanoparticle, nanotoxicity, biocompatibility, purity, synthesis, zebrafish

## Abstract

Gold nanoparticles (AuNPs) are increasingly used in biomedical applications, hence understanding the processes that affect their biocompatibility and stability are of significant interest. In this study, we assessed the stability of peptide-capped AuNPs and used the embryonic zebrafish (*Danio rerio*) as a vertebrate system to investigate the impact of synthesis method and purity on their biocompatibility. Using glutathione (GSH) as a stabilizer, Au-GSH nanoparticles with identical core sizes were terminally modified with Tryptophan (Trp), Histidine (His) or Methionine (Met) amino acids and purified by either dialysis or ultracentrifugation. Au-GSH-(Trp)_2_ purified by dialysis elicited significant morbidity and mortality at 200 µg/mL, Au-GSH-(His)_2_ induced morbidity and mortality after purification by either method at 20 and 200 µg/mL, and Au-GSH-(Met)_2_ caused only sublethal responses at 200 µg/mL. Overall, toxicity was significantly reduced and ligand structure was improved by implementing ultracentrifugation purifications at several stages during the multi-step synthesis and surface modification of Au-GSH nanoparticles. When carefully synthesized at high purity, peptide-functionalized AuNPs showed high biocompatibility in biological systems.

## 1. Introduction

Gold nanoparticles (AuNPs) are largely employed in the nanotechnology field in a variety of applications, mainly for their structural, electronic, optical and catalytic properties [1]. There is a growing interest around improving the synthesis method and purity of AuNPs, particularly those designed for medical applications [2,3]. The inherent optical resonance of AuNPs makes them especially useful in biomedical imaging applications [4]. The current study reports on the synthesis, uptake, and biocompatibility of spherical AuNPs with small peptide ligands as stabilizers designed for chelation and optical imaging applications.

Surface chemistry has been shown to play a role in the uptake and toxicity of AuNPs and the surface affinity of AuNPs enables the use of a large variety of inorganic and organic molecules as stabilizing ligands [5,6,7]. The formation of engineered AuNPs with custom surface ligands commonly involves a series of chemical reactions among reagents or precursors yielding colloidal suspensions that undergo stabilization by the addition of ligands or surfactants such as citrate or cetyl trimethylammonium bromide (CTAB) [1,8]. Often used in excess during the synthesis process, free or unbound stabilizers can remain after synthesis, requiring removal to prevent impacts on the stability, purity, and reactivity of the resulting nanoparticles [9].

Impurities (e.g., excess of ligands, starting reagents, conjugates) can contribute toxic effects and complicate understanding of inherent nanoparticle (NP) risk [10]. For example, unbound or free CTAB molecules present in solutions of CTAB-capped gold nanorods were found to be cytotoxic to HT-29 cells, while the gold nanorods without the capping agent were not [11]. Likewise, impurities in other NP types including carbon nanotubes have been reported as the source of toxic effects [10,12,13,14]. Overall, the presence of impurities originating from the synthesis process is not uncommon and is likely a confounding factor for interpreting biological responses [10,15]. Methods for reducing impurities in order to obtain pure NPs through improved synthesis methods are critical in the production of biocompatible nanomaterials.

A number of approaches have been employed to eliminate or mitigate the toxic effects imparted by impurities in nanomaterial suspensions including over-coating, surface ligand exchange, chelation of metal components, high-temperature thermal treatments, chromatography, field flow fraction, electrophoresis, dialysis, and diafiltration [11,16,17,18]. Despite the evidence suggesting that impurities must be removed from nanoparticle suspensions to avoid unwanted toxicity, these techniques are not regularly employed in many laboratories due the relative cost and time requirement for sample preparation [15,19].

Since the Au-GSH NPs are made with a 2-fold excess of GSH and a 10-fold excess of EDC/NHS, the excess tripeptides and coupling agents in the solution could compromise the intended ligand structure. Activated GSH ligands can react with the amine of Glu on free GSH and GSH-bound to AuNPs. Additionally, activated amino acids could form dipeptides as well as bind to the GSH. Although unbound and unwanted side products can be removed by dialysis or ultracentrifugation at the final stage of synthesis, the ligand structure on the NP surface can still be compromised by side-reactions with synthesis chemicals prior to removal. For this reason, an improved synthesis method that incorporates purification steps to produce high purity peptide-coated AuNPs with a well-defined ligand structure was a focus of the present study.

A major difference between the traditional synthesis methods described above and the improved synthesis strategy was the incorporation of additional ultracentrifugation purification steps during each stage of the multi-step modification of the AuNPs (Figure S1). In particular, before proceeding to the conjugation of the amino acid sequences to GSH-coated AuNPs, extensive ultracentrifugation of the precursor GSH-capped AuNPs was conducted before the coupling reaction with 1-(3-dimethylaminopropyl)-3-ethylcarbodiimide hydrochloride and N-hydroxysuccinimide (EDC/NHS) took place. Additional ultracentrifugation was employed after EDC/NHS coupling and addition of terminal amino acids (Table 1). We expect that the additional purification steps in this synthesis method minimized side reactions that could alter the intended ligand structure.

**Table 1:** Purification methods and synthesis strategies used to prepare GSH-capped AuNPs coupled with surface-bound amino acids.

AuNPs Descriptor	NP size (nm) *^a^*	Product (mM) *^b^*	Purification Technique	Synthesis Method
Au-GSH-(Trp)_2_-D	6.49 ± 2.88	1.82 × 10^−8^	Isopropyl wash/Dialysis purification with Milli-Q water	Standard chemical reduction of gold salts
Au-GSH-(His)_2_-D	7.99 ± 2.75	1.58 × 10^−8^	Dialysis purification with Milli-Q water	
Au-GSH-(Trp)_2_-U1	6.49 ± 2.88	9.84 × 10^−8^	Purification by ultracentrifugation with VivaSpin 20 column using basic water	Standard chemical reduction of gold salts
Au-GSH-(His)_2_-U1	7.99 ± 2.75	7.48 × 10^−8^		
Au-GSH-(Trp)_2_-U2	6.49 ± 2.88	1.22 × 10^−7^	Purification by ultracentrifugation with VivaSpin 20 column using basic water	Standard chemical reduction of gold salts
Au-GSH-(His)_2_-U2	7.99 ± 2.75	1.23 × 10^−7^		
Au-GSH-(Met)_2_-U2	7.29 ± 1.59	1.86 × 10^−7^		
Au-GSH-(Trp)_2_-U3	6.88 ± 1.76	2.91 × 10^−7^	Purification by ultracentrifugation with VivaSpin 20 column using 10 mM phosphate-buffer, pH = 8.0	Chemical reduction of gold salts using additional purification steps
Au-GSH-(His)_2_-U3	6.54 ± 1.78	1.54 × 10^−7^		
Au-GSH-(Met)_2_-U3	6.88 ± 1.98	1.92 × 10^−7^		

*^a^* Estimated based on TEM micrographs; *^b^* Amounts measured based on Optical Density of solutions.

GSH was selected as a capping agent to provide stability and water solubility to AuNPs via the carboxylate groups present within its structure. In addition, GSH has two available terminal carboxylic acid groups that can be exploited for coupling a wide-range of biologically relevant molecules useful in biomedical applications. GSH is found in high intracellular concentrations; thus, on the surface of the NP, this modified peptide sequence might serve as a good biomimic allowing for high NP uptake and potentially increasing bioavailability [20]. Following conjugation with GSH, NPs were further conjugated with sequences of amino acids (Trp, His or Met) by activating the terminal carboxyl groups of glutamic acid and glycine from GSH via the coupling reagents EDC and NHS [21,22].

Since NP surface charge is known to affect the uptake and toxicity of NPs, we selected amino acids that could differentially contribute to the resulting surface charge of particles and the stability of NPs in solution [23]. The nonpolar residues (Trp and Met) and a polar basic amino acid (His) were selected to improve Au-GSH NP stability and minimize nanoparticle-biological interactions. Trp and Met are both hydrophobic amino acids with neutral surface charge, and are therefore expected to have minimal nanoparticle-biological interactions. Conversely, histidine is a polar and basic amino acid that should display enhanced water-solubility, particle suspension stability, and biocompatibility.

The objectives of this study were to investigate how changes in surface chemistry, ligand composition, purification methods, and synthetic methods used in the preparation of peptide-coated AuNPs influence ligand structure, NP uptake, and biocompatibility in the embryonic zebrafish (*Danio rerio*). For these studies, we synthesized spherical GSH-coated AuNPs and modified the surface with one of three amino acids (Trp, His, or Met) to investigate how surface ligands influence NP uptake and biocompatibility. The effect of the non-nanoparticle components or impurities on toxicity and ligand structure were also investigated to determine the best way to purify conjugated NPs. Impurities were removed through dialysis or various ultracentrifugation techniques using filters with a specific molecular weight cutoff (MWCO) to separate the biological impacts of impurities themselves from those of unwanted side-reactions between synthesis chemicals and surface ligands.

## 2. Results and Discussion

The results of these studies show that synthesis and purification methods, as well as surface ligands, impact the overall structure and biocompatibility of Au-GSH NPs. High purity, biocompatible amino acid functionalized Au-GSH NPs can be produced when ultracentrifugation is used as a purification technique at each stage of a multi-step synthesis. The advantages of applying such techniques before and after the addition of each ligand in a multi-step synthesis include: (i) removing impurities from NP suspensions; (ii) producing more structurally stable ligands on the NP surface; and (iii) preventing side reactions during the coupling chemistry.

### 2.1. Nanoparticle Synthesis and Characterization

By monitoring the typical rapid color change in solutions and plasmon bands, the reduction of gold salts (Au^III^ → Au^I^ → Au^0^) and formation of colloidal gold was confirmed [24]. The water-solubility of stock solutions of Au-GSH-(X)_2_ (X = Trp, His, Met) at pH 8.0 indicate that the terminal amino acids are conjugated to GSH covalently attached to the NP surface. Transmission electron microscopy (TEM) was used to obtain information on primary NP sizes and morphologies (Figures S2a–c and S3a–c). All the NPs examined by TEM are spherical with core diameters ranging from 6 to 8 nm (Table 1). Further NP characterization was performed to obtain size distribution in aqueous solutions using dynamic light scattering (DLS) by measuring the intensity-weighted hydrodynamic diameter (Figures S2 and S3). The DLS results for particle size in basic water at pH 8.0 indicated that the unconjugated Au-GSH NPs (51 nm) had the largest hydrodynamicdiameter compared to the amino acid modified derivatives that had diameters of 39, 26 and 26 nm for Au-GSH-(Trp)_2_-U2, Au-GSH-(Met)_2_-U2, and Au-GSH-(His)_2_-U2, respectively (Figure S2d).

It should be noted that the TEM images represent the primary particle sizes as measurements were obtained under high vacuum conditions that require a dry sample preparation. In contrast, DLS size measurements were carried out in basic water at pH 8.0, and therefore are representative of hydrodynamic size and include homoaggregates resulting from particle interactions in an aqueous environment. In addition, the close nanoparticle-nanoparticle interactions observed with Au-GSH-(Trp)_2_-U2 could be due to π–π stacking interactions between the indole rings of Trp, influencing the DLS measurements. Although close nanoparticle-nanoparticle interactions are found in solution, the UV-Vis absorbance spectrum of the conjugated Au-GSH NPs showed a surface plasmon resonance (SPR) at λ_max_ at 522 nm (Figure S2e) indicating no significant red-shift of the SPR band resulting from aggregation of the materials after surface conjugation of terminal amino acids and purification. Similarly, no significant change in the diameter, hydrodynamic radius, and SPR band of the Au-GSH-(X)_2_-U3 was observed after multiple purification steps (Figure S3) [21].

### 2.2. Determination of Nanoparticle Purity

It is important to understand the potential for impurities to interfere with the efficiency of synthesis processes as well as alter the resulting product desired. This is particularly important in nanomaterial design where the surface is tailored for multi-functionality using multi-step synthetic strategies. In such cases, some of the reagents can undergo side reactions that impact the intended ligand structure on the NP surface. Although the detection and quantification of the types and levels of impurities following synthesis can be challenging [3], the results from thin layer chromatography (TLC), UV-Vis, fluorescence, and ^1^H NMR spectra illustrate the presence of only Au-GSH NPs with terminal amino acids conjugated onto the GSH ligand. That is, the presence of free GSH, amino acids, or other conjugated products after purification was not observed.

**Figure 1:**
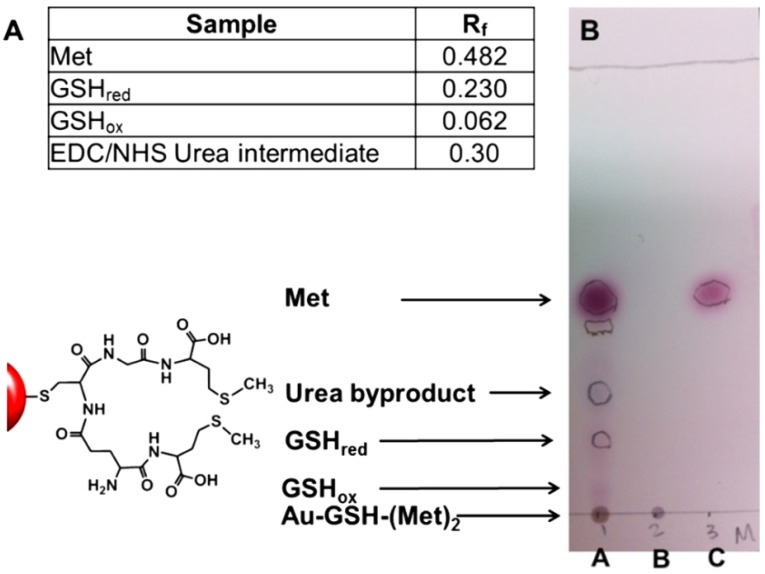
Thin layer chromatography (TLC) determination of Au-GSH-(Met)_2_-U2 purity after ultracentrifugation. (**A**) R_f_ values for compounds listed on the TLC; (**B**) TLC plate of Au-GSH-(Met)_2_-U2: *Lane A* before purification, *Lane B* after purification by ultracentrifugation, and *Lane C* of free Met ligand in butanol/acetic acid/H_2_O (12:3:5) solvent system.

Both TLC and ^1^H NMR spectroscopy were used to assess the amount of excess precursor molecules or impurities in the samples and permeates after extensive washing by several methods. TLC of AuNP samples was performed using a mixture of butanol/acetic acid/H_2_O (12:3:5) followed by spraying with ninhydrin. Figure 1A lists the retention factor (R_f_) values of individual spots that confirm the presence of unconjugated products from the synthesis of Au-GSH-(Met)_2_-U2 such as free methionine, reduced GSH (GSH_red)_, oxidized GSH (GSH_ox_), and a urea byproduct from the coupling reaction. A representative TLC of the unpurified and purified Au-GSH-(Met)_2_ NPs is shown in Figure 1B. Lane C consists of pure Met, while Lane A and B is spotted with unpurified and purified Au-GSH-(Met)_2_-U2 NPs respectively. Lane B showed only one spot indicative of the anionic AuNPs, with no spots characteristic of free amino acids, coupling agents or conjugated byproducts present on the silica plate. In contrast, Lane A shows several distinguishable spots in the unpurified Au-GSH-(Met)_2_-U2 NPs with R_f_ values corresponding to unconjugated Met, GSH_ox_, GSH_red_, and the urea byproducts. Although not confirmed, minor spots observed suggest that there are trace amounts of conjugated GSH-Met products. Similarly, spots corresponding to free amino acids, the urea byproduct from the EDC/NHS coupling, coupled GSH_red_, and GSH_ox_ were also observed for Au-GSH-(Trp)_2_-U2 and Au-GSH-(His)_2_-U2 derivatives (Figure S6). Most importantly, this demonstrates that TLC is an effective tool for determining the purity of peptide-stabilized AuNPs.

**Figure 2:**
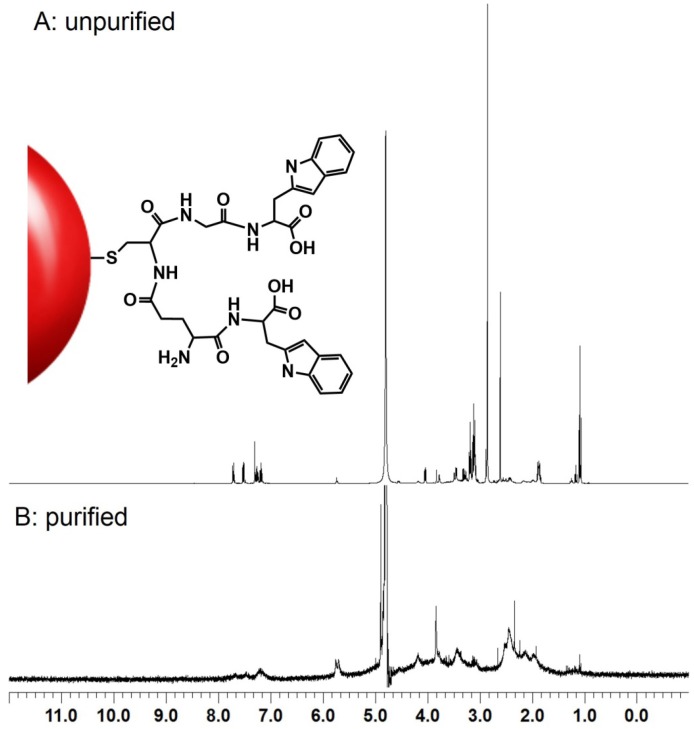
Representative ^1^H NMR spectra of (**A**) unpurified and (**B**) purified Au-GSH-(Trp)_2_-U2 nanoparticles in H_2_O at 25 °C.

^1^H NMR spectra was taken of the unpurified and purified Au-GSH-(X)_2_-U2 (X = Trp, His, Met) samples. A representative ^1^H NMR spectra of Au-GSH-(Trp)_2_-U2 NPs shown in Figure 2, illustrates a substantial difference in the ^1^H NMR spectra of unpurified and purified peptide-stabilized NPs. Specifically, the ^1^H NMR spectra of unpurified Au-GSH-(Trp)_2_-U2 exhibited a complicated spectrum with sharp signals that correspond to protons on the amino acid-modified GSH ligands, precursor molecules, and other byproducts from the synthesis (Figure 2A). The sharpness of the signal in the spectra is consistent with free ligands present in the sample (Figure 2A). In contrast, the ^1^H NMR spectra of a purified concentrated sample of Au-GSH-(Trp)_2_-U2 showed significant line broadening making it difficult to assign any proton signals (Figure 2B), indicating that the free ligand concentration is below the NMR detection limit. That is, it confirms that the AuNPs are highly pure with minimal free ligands, consistent with previous results for other high purity nanomaterials [17].

Results from UV-Vis and fluorescence spectroscopy also confirmed the purity of the nanomaterials observed by TLC and ^1^H NMR spectroscopy. Since Trp, His, and Met ligands absorb in the UV-Vis region (200–300 nm), analyses were performed on 1 mL of concentrated permeate samples collected by ultracentrifugation of the final wash. No characteristic absorption in this 200–300 nm region was observed indicating all free amino acids were removed. In addition, while the fluorescence spectra of purified Au-GSH-(Trp)_2_-U2 NPs did confirm that Trp was conjugated to the AuNPs by observance of an significantly quenched emission band at λ_max_ 365 nm for Trp upon excitation at 280 nm; the fluorescence spectra of the concentrated 1 mL permeate showed no evidence of Trp. However, ample free Trp in the permeate was observed by fluorescence after the first wash of the Au-GSH-(Trp)_2_-U2.

The synthesis and purification methodologies used can impact the purity as well as the coupling efficiency. For example, since the coupling chemistry in D and U1-2 is performed in the presence of excess EDC/NHS one possible side reaction that could occur is competition between the amine of GSH and amino acids for the activated carboxylic acids. That is GSH-GSH and dipeptide (e.g., Trp-Trp) coupling could lower the conjugation efficiency. Additionally, since the reaction is also done in the presence of excess amino acids these can become activated and couple to the amine as well as carboxylic acid groups of GSH to form GSH-(X)_3_. Using UV-Vis spectroscopy we were able to estimate that the coupling efficiency of Au-GSH-(Trp)_2_-U2 was ~100% (supplemental information). This theoretical assumption is based on a Au:GSH ratio of 1:1 and GSH:Trp ratio of 1:2 on the AuNP surface. However, it is possible that there is less GSH on the surface than estimated and that Trp is coupling to the amine of GSH to form a GSH-(X)_3_ species. While we were not able to confirm the ligand structure of Au-GSH-X_2_-U2 NPs, we were able to confirm the composition of the ligand structure of Au-GSH-X_2_-U3 NPs by ^1^H NMR spectroscopy [21]. Cyanide and HCl etched samples of Au-GSH-X_2_-U3 in methanol showed the presence of only free GSH-X_2_ in samples prepared by U3 ligands [21]. This demonstrates that U3 method can limit unwanted side reactions for 100% coupling.

### 2.3. Impact of Purity and Synthesis Method on Toxicity

**Figure 3:**
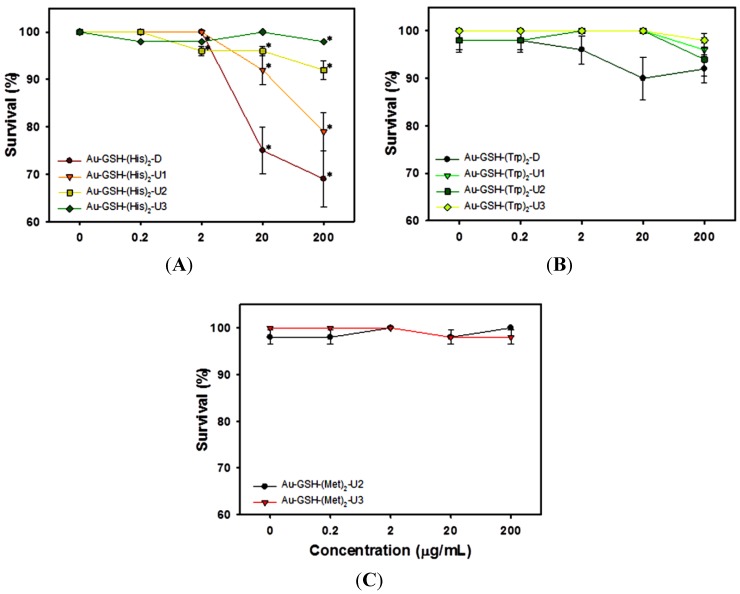
Survival rates for embryonic zebrafish exposed to varying concentrations of Au-GSH-(X)_2_ (X = Trp, His, and Met) nanoparticles. Survival measured at 120 hpf for AuNPs with (**A**) His; (**B**) Trp; (**C**) Met. Results are presented as mean ± SEM. Asterisks indicate significant differences from control (untreated, concentration = 0) embryos (*p* ≤ 0.05, *n* = 48).

**Figure 4:**
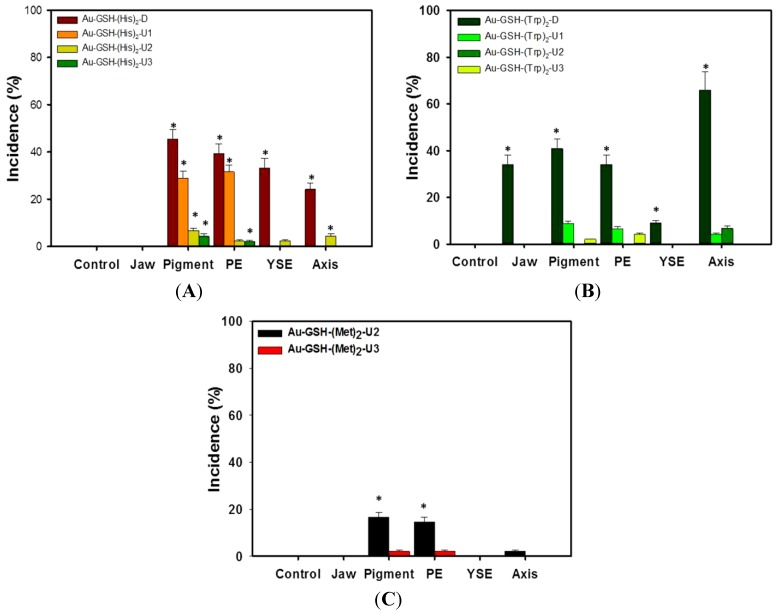
Incidence of sublethal effects in zebrafish embryos after 5 days of exposure to 200 µg/mL Au-GSH nanoparticles conjugated with (**A**) His; (**B**) Trp; or (**C**) Met. Data on malformations are presented as mean ± SEM (*n* = 48). Asterisk indicates significant difference exists in the percent incidence *vs.* control (untreated) embryos (*p* ≤ 0.05, *n* = 48).

Different purification techniques at different stages of the synthesis were used to remove impurities from NP suspensions in order to limit side reactions with activated free GSH or amino acids that could complicate the ligand structure and impact NP biocompatibility. Nanoparticles purified by dialysis (D), ultracentrifugation (U1-2) and an improved synthesis strategy incorporating membrane ultracentrifugation at each stage of the multi-step reaction (U3) showed significant differences in both mortality and sub-lethal impacts on developing fish. In general, toxicity was inversely correlated with the amount of purification techniques used during synthesis of the AuNPs (Figure 3 and Figure 4). The Au-GSH NPs without amino acid conjugation had poor stability in the fishwater test solution when highly purified and thus were not included in the toxicity studies.

Highly purified and carefully synthesized Au-GSH-(X)_2_ (X = Trp, His, Met) NPs were found to be biocompatible at concentrations as high as 200 µg/mL when the improved synthesis strategy (U3) was implemented (Figure 3 and Figure 4). Our findings suggest that NPs synthesized using a standard chemical reduction and purified by ultracentrifugation (U2) to remove non-nanoparticle components still produce AuNPs that elicit toxic responses suggesting that differences in the ligand structure are influencing biocompatibility (Figure 3). These differences might be due to intentional design of the Trp, His, or Met modified GSH ligand structure on the surface or compromised ligand structure from competing side reactions during synthesis.

Gold NPs with His surface functionalities induced higher overall embryonic toxicity (both mortality and morbidity) than those with either Trp or Met ligands prepared by the same method (Figure 3 and Figure 4). AuNPs conjugated with His surface ligands showed the highest toxicity with minimal purification (D, U1; Figure 3A). Au-GSH-(His)_2_ particle toxicity was mitigated in U3 NPs at all doses except the highest dose tested (200 µg/mL; Figure 3A and Figure 4A), while other purification techniques resulted in significant toxicity at doses as low as 2 µg/mL. These results indicate that although the same technique was used to purify the peptide-capped AuNPs, the resulting structures of these terminal sequences could also play a role in their biocompatibility.

In addition to impurities, another potential explanation for differential biological responses to the AuNPs is the composition of the ligand structure. In samples where ultracentrifugation was used at either the final stage (U1-2) or during each stage of the multi-step synthesis (U3) there were minor sublethal effects. Although the synthetic scheme (Figure S1) is the same for all NPs, we were not able to confirm the final ligand structures on the AuNP surfaces synthesized with no additional purification steps (D, U1-2). While this is a limitation of the present study, we believe that excess ligands and the lack of additional purification steps during synthesis led to unwanted coupling of side products and uncontrolled growth of ligand sequences on AuNP surfaces. More specifically, excess activated GSH and amino acids in the first and last steps of the synthesis can compromise the final ligand structure by binding to each other and the surface of GSH-bound AuNPs.

The terminal acid residues also played a role in the unique biocompatibility of the Au-GSH NPs. The nonpolar hydrophobic Trp and Met residues as well as polar basic His residues of the Au-GSH-(X)_2_ (X = Trp, His, Met) NPs may mask the delivery of AuNPs to the target and reduce charge-dependent interactions in the exposure environment. This is similar to peptide-capped silver NPs with terminal positively charged lysine or neutral serine peptides that impart more biocompatibility compared to peptides with negatively charged glutamic acid residues [23].

The survival of embryos exposed to Au-GSH NPs with either Met or Trp surface ligands was high and did not differ from control with any of the purification techniques (Figure 3B,C). Trp exposed embryos (200 µg/mL) had significantly higher occurrences of pericardial and yolk sac edema, curved body axis, jaw malformations and pigmentation abnormalities when purified by dialysis, that did not occur following any of the ultracentrifugation methods (Figure 4B). With the exception of jaw malformations that were not noted in the His functionalized particles, the sublethal impacts of His NPs were similar to the Trp conjugated NPs, except with His derivatives the sublethal toxicity was not mitigated by U3 techniques (Figure 4A). Au-GSH-Met NPs were found to induce significant pericardial edema and pigmentation abnormalities at 200 µg/mL with U2 type purification, but those impacts were removed when the U3 technique was used to purify the NPs (Figure 4C). Although mortality was greater with Au-GSH-(His)_2_-D than the others (Figure 3), some malformations were commonly observed among the NPs purified by dialysis (D), ultracentrifugation (U1-2) and improved synthesis incorporating ultracentrifugation in each stage of the multi-step reaction (U3); those include pericardia edema, yolk sac edema, over-pigmentation, jaw malformations and curved body axis (Figure 4).

The lowest toxicity observed in NPs, measured by mortality and morbidity, to the highest toxicity can be ranked as: Au-GSH-(Trp)_2_-U3; -(His)_2_-U3; -(Met)_2_-U3 >> Au-GSH-(Trp)_2_-U2; -(His)_2_-U2; -(Met)_2_-U2 > Au-GSH-(Trp)_2_-U1; -(His)_2_-U1 > Au-GSH-(Trp)_2_-D; -(His)_2_-D. The observed reduction of toxicity was most likely due to the removal of impurities such as unconjugated amino acids, activated GSH/amino acids, unbound reduced/oxidized conjugates, and EDC/NHS byproducts.

Overall, ultracentrifugation was found to be the most efficient technique for purification because it was best able to reduce the amount of impurities, and ultimately resulted in an overall lowered toxicity to embryos. Incorporating additional ultracentrifugation purifications at each stage of the multi-step synthesis (U3) increases biocompatibility compared to singular purification at the final stage of the synthesis (D, U1, U2). This method of implementing several ultracentrifugation steps in a multi-step reaction also reduces the non-nanoparticle components with the potential to significantly alter the overall composition of surface ligands through side reactions; which, in turn, can alter the toxicity of the final product.

### 2.4. Nanoparticle Uptake

**Figure 5:**
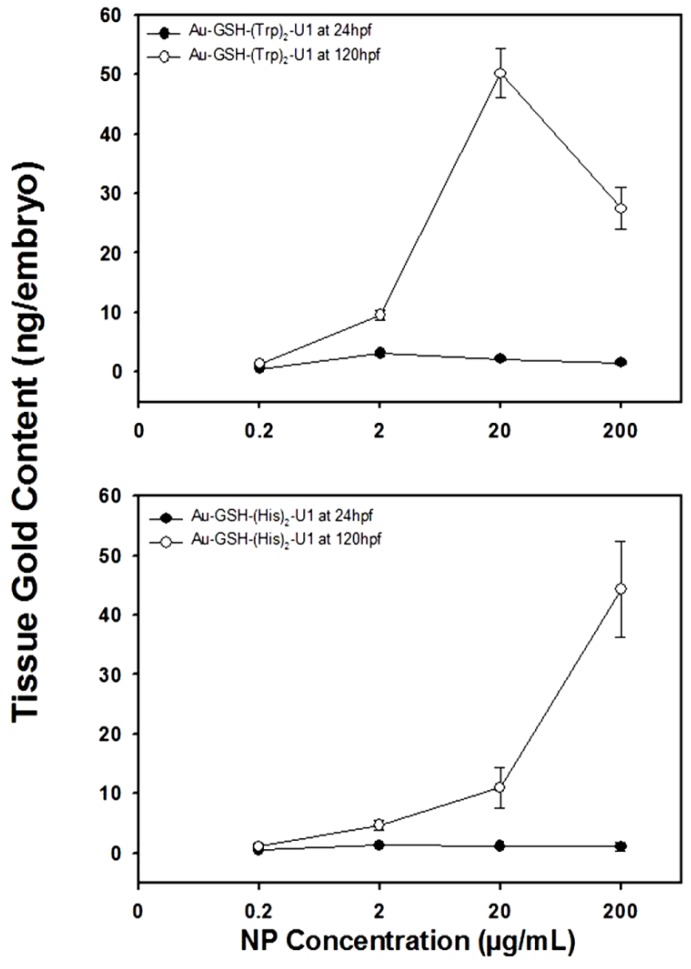
Uptake of AuNPs containing (**A**) Au-GSH-(His)_2_-U1 or (**B**) Au-GSH-(Trp)_2_-U1, both purified by ultracentrifugation as measured by INAA in zebrafish at 24 and 120 hpf. Data are presented as mean ± STDV of three independent samples (*n* = 3). Asterisk indicates significant difference exists in gold content compared to untreated embryos (*p* ≤ 0.05, *n* = 48).

We used Instrumental Neutron Activation Analysis (INAA) to compare Au-GSH-(His)_2_-U1 and Au-GSH-(Trp)_2_-U1 NP uptake into embryos and observed a dose-dependent increase in tissue gold concentration. Although INAA reports the total amount of gold, with no distinction in chemical states, its high detection sensitivity makes INAA a viable alternative for reporting the presence of metal nanomaterials in organisms [6]. The uptake of Au-GSH NPs by zebrafish embryos was quantified by INAA at 24 and 120 hpf for Au-GSH-(His)_2_-U1 and Au-GSH-(Trp)_2_-U1 (Figure 5). The tissue content of Au-GSH-(His)_2_-U1 NPs at 24 hpf was similar among all test concentrations. However, at 120 hpf the gold content in the embryonic tissues began increasing in a concentration-dependent manner with significant increases at starting dosages above 2 µg/mL (Figure 5A). Similar results were observed for the tissue content of Au-GSH-(Trp)_2_-U1 with the exception of a slight decrease in tissue concentration at the highest dose, possibly due to the observed AuNP agglomeration at the highest test concentration (data not shown). This is consistent with DLS studies where the Au-GSH-(Trp)_2_-U1 have close nanoparticle-nanoparticle interactions in solution at high concentrations and explains the decreased tissue uptake. INAA was also used to determine the gold content in the testing solutions alone (without embryos), and no significant differences between nominal and measured concentrations were found (Figure S8). These results indicate that the same mass of NPs were available per embryo during the exposure.

Overall, the dose-dependent uptake of biocompatible peptide-capped AuNPs in the zebrafish demonstrates the utility of these NPs in biomedical applications such as optical imaging [25]. Other studies have utilized folic acid and fluorescein dyes coupled to GSH-capped AuNPs in a similar approach for targeting and imaging cancer cells [22]. Amino acid coupling may impart not only higher uptake, but also the longer pentapeptide sequence imparts greater stability through increased hydrogen bonding interactions with water than the shorter chained tripeptide GSH ligand alone. The incorporation of terminal amino acids in the ligand design also keeps two carboxylic acids residues of the new amino acids open for further coupling chemistry for longer sequences and coupling to other biomolecules for specific applications. That is, conjugation chemistry and purification method implemented (U3) can be used to tailor the surface of the NPs with a variety of biomolecules such as targeting ligands, drugs, and imaging agents.

## 3. Experimental Section

### 3.1. Materials

Chloroauric acid (HAuCl_4_·*x*H_2_O) was purchased from Strem Chemicals, Inc. (Newburyport, MA, USA) and reduced L-glutathione (GSH) was purchased from Sigma-Aldrich Chemical Co. (St. Louis, MO, USA). Sodium borohydride and TLC Silica Gel1B plates were from J. T. Baker and Co. (Boston, MA, USA), N-hydroxysuccinimide 98% (NHS) was from Acros and 1-(3-dimethylaminopropyl)-3-ethylcarbodiimide hydrochloride (EDC) was purchased from TCI America (Portland, OR, USA). All chemicals were used as received. High purity particle free water was obtained from a Milli-Q water purification system (EMD Millipore, Billerica, MA, USA). Thin layer chromatography (TLC) was performed using Baker-Flex using 5 cm × 20 cm Silica Gel 1B plates with a 200 µm analytical layer and using a mixture of butanol/acetic acid/H_2_O (12:3:5) as the mobile phase.

UV-Vis spectra were recorded in water using an Ocean Optics USB4000 UV-Visible-NIR spectrophotometer with a 1.0 cm path length quartz cell. Infrared spectra were recorded on a Thermo Scientific Nicolet iS10 Smart iTR and fluorescence measurements were performed with a PTI spectrophotometer using Felix32 software. Measurements were taken using a quartz cell at an excitation of 280 nm and emission of 365 nm with a 4 nm bandpass on both monochromators. Dynamic light scattering (DLS) measurements were performed with an LB-550 particle size analyzer (Horiba Co. Ltd., Fukushima, Japan). Transmission Electron Microscopy (TEM) data were acquired on a Tecnai F-20 FEI microscope at an acceleration voltage of 200 kV using a CCD detector. Samples were prepared by drop casting dilute solutions of nanoparticles onto carbon-coated (300 Å) Formvar films on copper grids (Ted Pella Inc., Redding, CA, USA) and allowing the samples to air dry overnight before imaging.

### 3.2. Syntheses of Au-GSH-(X)_2_ (X = Trp, Met, His) Nanoparticles

The synthesis of the Au-GSH NPs was modified from a similar procedure [26]. Briefly, HAuCl_4_·*x*H_2_O (0.025 g, 0.074 mmol) and reduced GSH (0.045 g, 0.147 mmol) were dissolved in 10 mL of H_2_O and stirred vigorously for 30 min. Immediately, the yellow-gold color disappears and after 30 min a cloudy colorless solution appears. A freshly prepared aqueous solution of NaBH_4_ (0.028 g, 0.735 mmol in 5 mL of H_2_O) was added one drop/sec under vigorous stirring until the solution changed from a brown to final maroon-red color that was stirred overnight at 25 °C. To these NPs EDC (0.084 g, 0.438 mmol) was added followed by NHS (0.051 g, 0.441 mmol) under vigorous stirring. The reaction was left to stir for 1 h before 0.294 mmol terminal amino acid (Trp = 0.060 g, His = 0.046 g, or Met = 0.050 g) were added followed by additional stirring over 24 h. This was followed by purification by either ultracentrifugation or dialysis. Ultracentrifugation was performed at the end of the synthesis (U2) or prior to and after amino acid coupling (U3) with 10 mM sodium phosphate buffer pH 8 (10 mL × 45 times) with a Thermo Scientific, Sorvall ST 40R at 4700 rpm using Sartorius Stedim Biotech ultracentrifuge concentrators with a PES membrane (Vivaspin 20, MWCO = 10 K). Dialysis was performed with Spectra/Por dialysis membrane with a MWCO of 1 K in basic water (pH 8.0). To remove unbound salts: coupling reagents, amino acids, and GSH. The purity of the material was determined by TLC using a butanol/acetic acid/H_2_O (12:3:5) mixture.

### 3.3. Exposure Suspensions

Stock solutions of AuNPs were dispersed in fishwater comprised of 0.26 g/L Instant Ocean salts (Aquatic Ecosystems, Apopka, FL, USA) in reverse osmosis (RO) water adjusted to pH 7.2 ± 0.2 with sodium bicarbonate. Test NP solutions were freshly prepared at 0.2, 2.0, 20 and 200 µg/mL; control solutions contained fishwater alone.

### 3.4. Embryonic Zebrafish Assay

The exposure protocol has been previously described in Truong *et al.*, 2011 [27]. Briefly, zebrafish (*Danio rerio*) embryos were collected from group spawns of wild-type D5 fish housed at the Sinnhuber Aquatic Research Laboratory (Oregon State University, Corvallis Oregon). Twenty four embryos per treatment (2 replicates, *n* = 48) were placed individually in clear 96-well plates containing NP suspensions (0 to 200 µg/mL) at 8 h post-fertilization (hpf). Plates were sealed with Parafilm and kept under a 14:10 h light:dark photoperiod at 26.8 °C for 5 days. Embryos were assessed at 24 hpf for mortality, developmental progression, and spontaneous movements; then at 120 hpf for mortality, morphology (body axis, eye, snout, jaw, otic vesicle, notochord, heart, brain, somite, fins, yolk sac, trunk, circulation, pigment, swim bladder) and behavioral response (motility). The percent incidence of each endpoint was calculated for each treatment. Experiments were performed in accordance with all national and local guidelines and regulations.

### 3.5. Embryonic Uptake of AuNPs

Instrumental Neutron Activation Analysis (INAA) was used to determine the concentration of elemental gold taken-up by embryos exposed to Au-GSH-(Trp)2-U1 and Au-GSH-(His)2-U1 NPs. Three exposed embryos from each NP treatment were collected at each exposure concentration and thoroughly washed with Milli-Q water prior to being transferred to polyethylene radiation vials. Embryos were dried at 35 °C before INAA analysis. Aliquots of exposure solutions, fishwater alone and embryos exposed to only fishwater at 24 and 120 hpf were also analyzed to control for any background metals.

### 3.6. Data Analysis

Statistical analyses were conducted using SigmaPlot 12.2 (Systat Software, San Jose, CA, USA). Analysis of variance was first used to ensure all replicates could be pooled before the nonparametric Fishers Exact test was used to determine if the incidence of endpoints in the replicate fish (*n* = 48) varied significantly from control embryos (exposed to fishwater alone) or varied significantly between treatments [28].

## 4. Conclusions

Many of the differences in toxicity observed in these studies suggest that the presence of impurities can impact the toxicity, function, and properties of nanomaterials, similar to other studies [3,12]; however, very little has been reported on how impurities can lead to unwanted coupling reactions resulting in changes of the structure of the surface ligands. Here, we report that this scenario can occur and can be associated with increased toxicity.

Our results confirm the overall biocompatibility of highly purified terminally modified GSH-coated AuNPs for imaging and metal chelation applications. Here, we have presented a comparative toxicological analysis of a series of GSH-coated AuNPs with terminal ligands of nonpolar hydrophobic and polar basic amino acids. The biological responses of such NPs were strongly influenced by the purification method and the incorporation of additional ultracentrifugation steps during each stage of a multi-step synthesis. Previous studies have provided support for the biocompatibility of Au-GSH NPs in other living systems at similar test concentrations with Au-GSH particles of other sizes [29,30,31]. The removal of impurities and incorporation of additional ultracentrifugation during each stage of a multi-step synthesis as part of a synthetic strategy, regardless of ligand composition, can have a dramatic impact on the toxicity of the NPs. Furthermore, the incorporation of ultracentrifugation before and after the coupling reaction with the amino acid sequences allowed for a more efficient conjugation that translated into an enhanced biocompatibility.

## Supplementary Materials

Supplementary File 1
