# Supplementary material for: The Impact of Surface Ligands and Synthesis Method on the Toxicity of Glutathione-Coated Gold Nanoparticles
Source: Nanomaterials (Basel). 2014 May 12;4(2):355–71. doi: 10.3390/nano4020355 (PMC4512953; doi:10.3390/nano4020355)
Supplement: Supplementary File 1 [file nanomaterials-04-00355-s001.pdf]

Supporting Information

Figure S1. Experimental design for the GSH-coated AuNP studies.

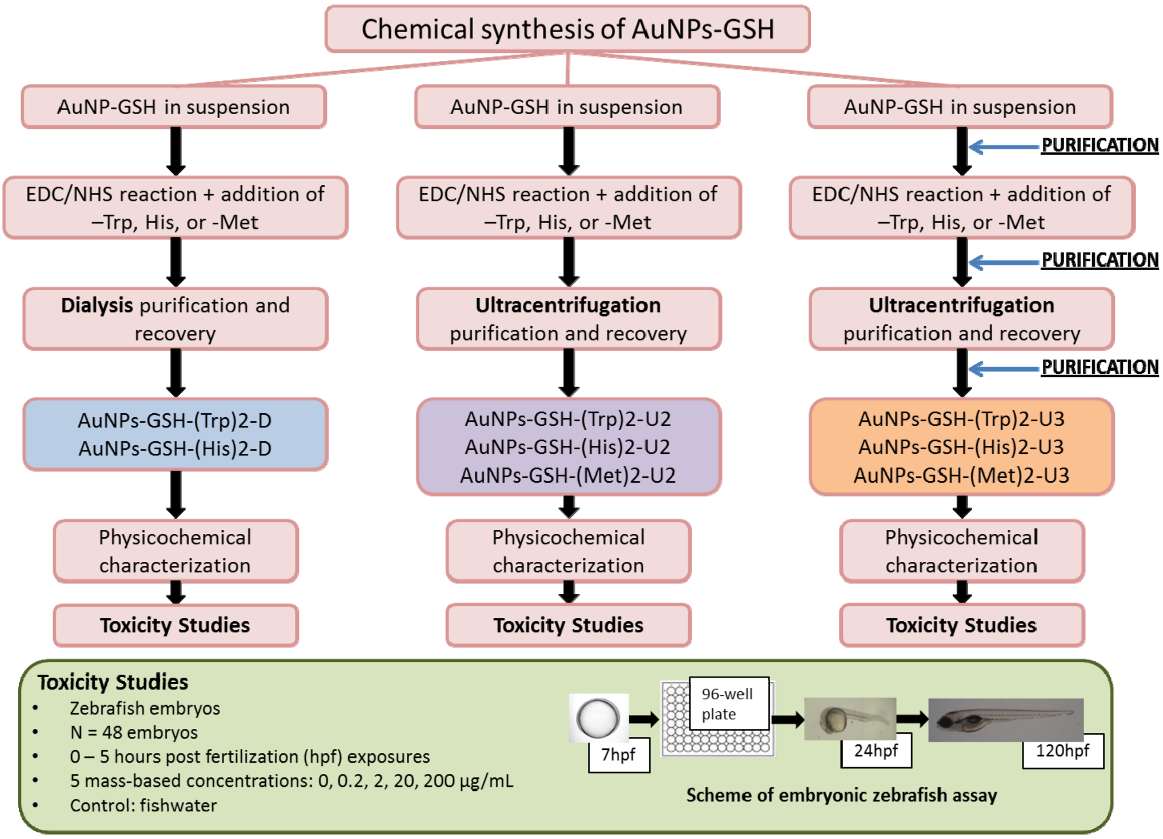

Figure S2. Physicochemical characterization of the U2 nanoparticles. TEM images of Au-GSH NPs with (**a**) GSH-Trp_2-U2; (**b**) GSH-Met_2-U2; and (**c**) GSH-His_2-U2. Scale bar = 20 nm; (**d**) Hydrodynamic diameters of AuNPs with (A) GSH, (B) GSH-Trp_2-U2, (C) GSH-Met_2-U2, and (D) GSH-His_2-U2; (**e**) UV-Vis spectra of AuNPs with (A) GSH, (B) GSH-Trp_2-U2, (C) GSH-Met_2-U2, and (D) GSH-His_2-U2.

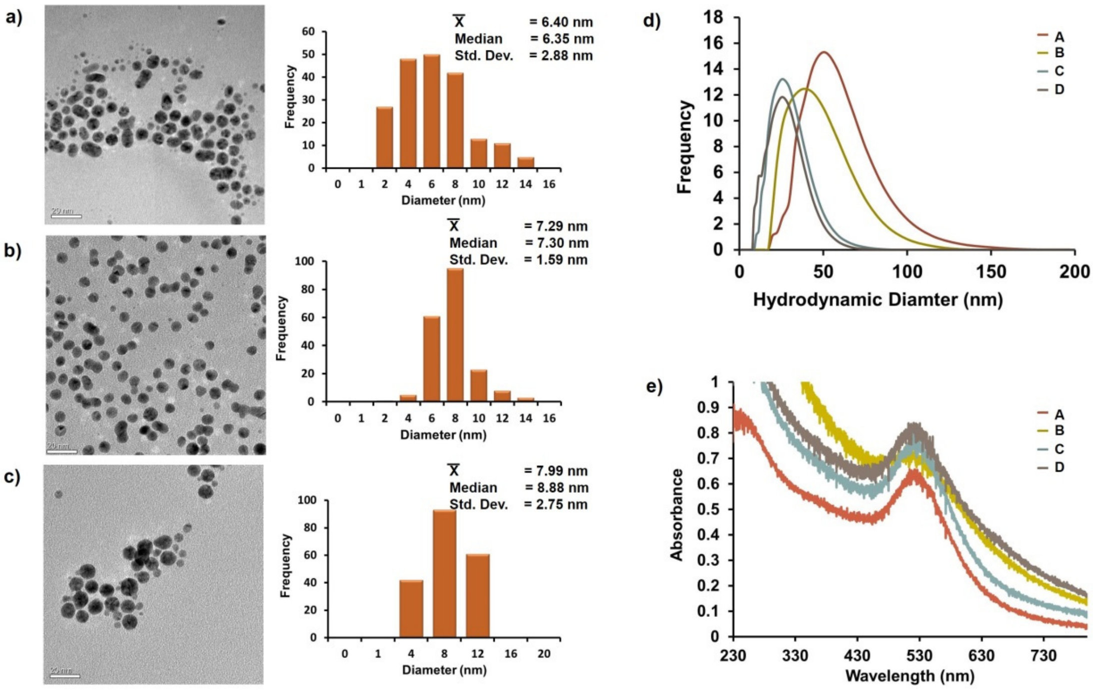

Figure S3. Physicochemical characterization of the U3 nanoparticles. TEM images of Au-GSH NPs with (**a**) GSH-Trp_2-U3; (**b**) GSH-Met_2-U3; and (**c**) GSH-His_2-U3. Scale bar = 20 nm; (**d**) Hydrodynamic diameters of AuNPs with (A) GSH, (B) GSH-Trp_2-U3, (C) GSH-Met_2-U3, and (D) GSH-His_2-U3; (**e**) UV-Vis spectra of AuNPs with (A) GSH, (B) GSH-Trp_2-U3, (C) GSH-Met_2-U3, and (D) GSH-His_2-U3.

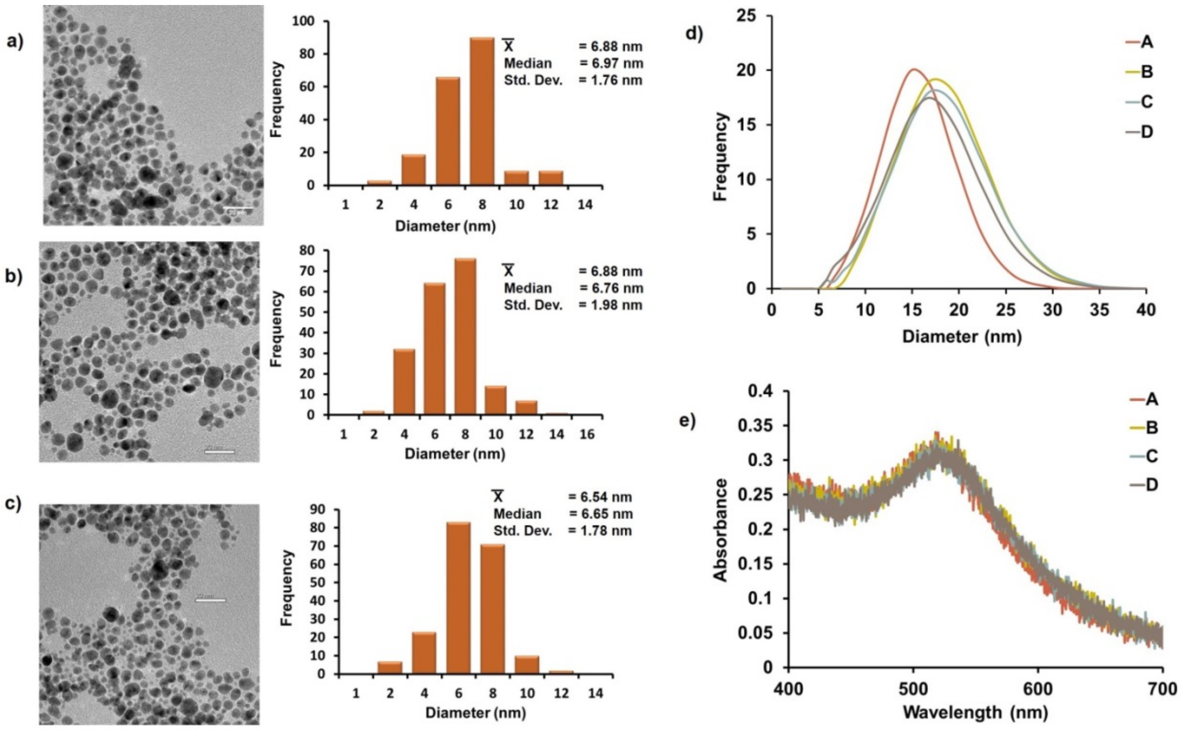

Figure S4. Au-GSH-$(Trp)_2$ nanoparticles (O.D = 0.199, λ_{max} = 520 nm) in the presence of 11.8 mM of KCN at (**a**) 0 h; (**b**) 1 h; and (**c**) 48 h in basic water at pH 8.0.

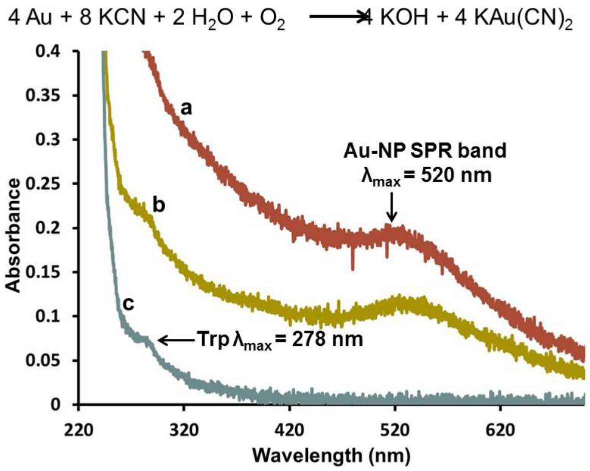

Supplemental Information on Ligand Coupling Efficiency

To determine the coupling efficiency of the amino acids to GSH it was necessary to determine the amount of GSH ligands on the nanoparticle surface. The synthesis of the nanoparticle in the presence of excess thiol was done in 1:2 ratio of Au:GSH. To calculate the amount of ligand coverage on the nanoparticle surface of a certain diameter (D) the optical density of the stock solutions was converted to a concentration using Beer's Law and Equation (1), where k and a are 3.32 and 10.8, respectively, based on a study of similar nanoparticles by Liu *et al*. 2007 [1].

$$\ln \varepsilon = k \ln D + a \quad (1)$$

The number of entities (N) per mL was determined from Equation (2) using Avogadro's number, N_A, and the concentration of the nanoparticles, C:

$$N = N_A C \quad (2)$$

For example, with the Au-GSH-$(Trp)_2$ nanoparticles approximately 798 gold atoms are estimated to be on the surface. Therefore, assuming at 1:1 binding ratio of Au:GSH an equivalent number of GSH molecules are required to cover a nanoparticle with an average diameter of 6.4 nm. In addition, for each GSH molecule there are two terminal carboxylic acid groups, therefore, 1596 carboxylic acid groups are available for coupling with Trp amino acids on the surface of the nanoparticles. Theoretically, based on the optical density of a solution containing 1 mL Au-GSH-$(Trp)_2$ nanoparticles (O.D. = 0.199) with an average diameter of 6.4 nm there are approximately 13.6 nmoles of Trp molecules conjugated to each carboxylic acids on the GSH tripeptide.

To confirm the amount of Trp actually coupled to the GSH ligand on the gold nanoparticle surface, a 1 mL Au-GSH-$(Trp)_2$ nanoparticles (O.D. = 0.199) was etched with cyanide (11.8 mM) to decompose the gold core. Cyanide, a well-known etchant that oxidizes Au^0 to Au^I was used etch the nanoparticles so that the Trp absorption could be seen which was previously hidden by the SPR band. Within 1 h of cyanide addition a 40% drop in the optical density of the SPR band was observed. The slow rate of cyanide etch suggests that gold nanoparticle surface is covalently passivated by the thiolated peptide ligands. The UV-Vis spectra taken after 48 h of a colorless solution showed an absorption at 278 nm characteristic for Trp. Using the optical density (0.075) and the molar absorption coefficient (5579 $M^{-1} \cdot cm^{-1}$) the concentration of Trp was determined to be 13.4 μM, which is equivalent to 14.0 nmoles of Trp after cyanide decomposition. The number of moles of Trp theoretically calculated using the diameter of the nanoparticle core and optical density of Au-GSH-$(Trp)_2$ is almost equivalent to the number of moles experimentally determined after cyanide decomposition. Therefore, Trp coupling to GSH occurs with 100% efficiency in a 1:2 GSH:Trp ratio.

Figure S5. Scheme of synthesis of GSH-coated gold nanoparticles with surface-bound nonpolar amino acids.

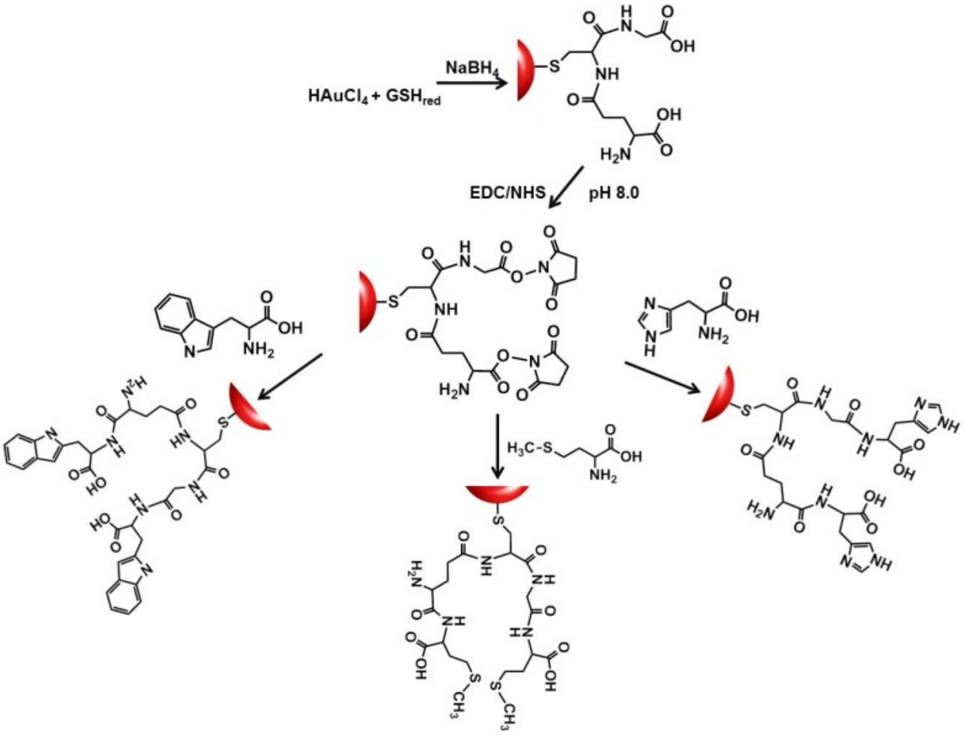

Figure S6. Determination of purity of AuNPs-GSH-(Trp)$_2$-U2 and AuNPs-GSH-(His)$_2$-U2 NPs by ultracentrifugation.

A

Sample	Trp	Met	His	GSH	GSSG	EDC/NHS Urea intermediate
R_f	0.574	0.482	0.096	0.23	0.062	0.304
R_f Au-GSH-(Trp)$_2$	0.574			0.26	0.065	0.333
R_f Au-GSH-(Met)$_2$		0.482		0.26	0.065	0.333
R_f Au-GSH-(His)$_2$			0.096	0.24		0.30
R_f Au-GSH					0.05	
Average R_f	0.574	0.482	0.096	0.247	0.06	0.317

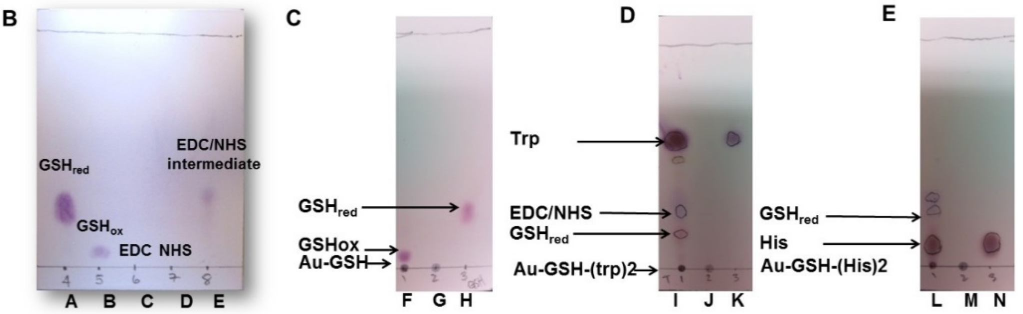

F

LANES	SAMPLE	LANES	SAMPLE
A	GSH$_{red}$	J	purified Au -GSH(Trp)$_2$
B	GSH$_{ox}$	K	Trp
C	EDC	L	unpurified Au -GSH(His)$_2$
D	NHS	M	purified Au -GSH(His)$_2$
E	EDC/NHS	N	His
F	Unpurified Au-GSH		
G	Purified Au-GSH		
H	GSH$_{red}$		
I	Unpurified Au-GSH(Trp)$_2$		

Figure S7. Incidence of sublethal effects of AuNPs-GSH-$(His)_2$ nanoparticles. Malformations observed in embryos exposed to 20 µg/mL of AuNPs-GSH-$(His)_2$ NPs. Data are presented as mean ± SEM (n = 24, two replicates). (*) indicates significant difference exists in percent incidence among the same batch of NPs ($p < 0.01$); (**) indicates significant difference exists in percent incidence across the batches of NPs ($p < 0.01$).

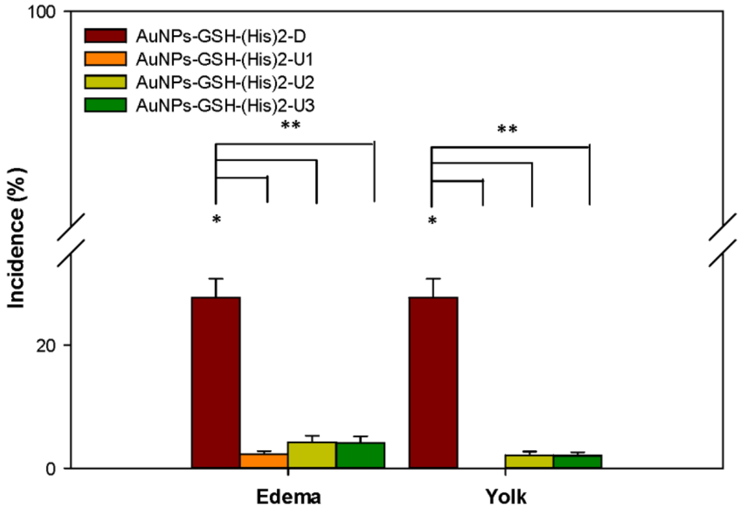

Figure S8. Nominal and measured concentrations of (**A**) AuNPs-GSH-$(His)_2$-U1 and (**B**) AuNPs-GSH-$(Trp)_2$-U1 nanoparticles. Data are presented as mean ± STDV of three independent samples (n = 3).

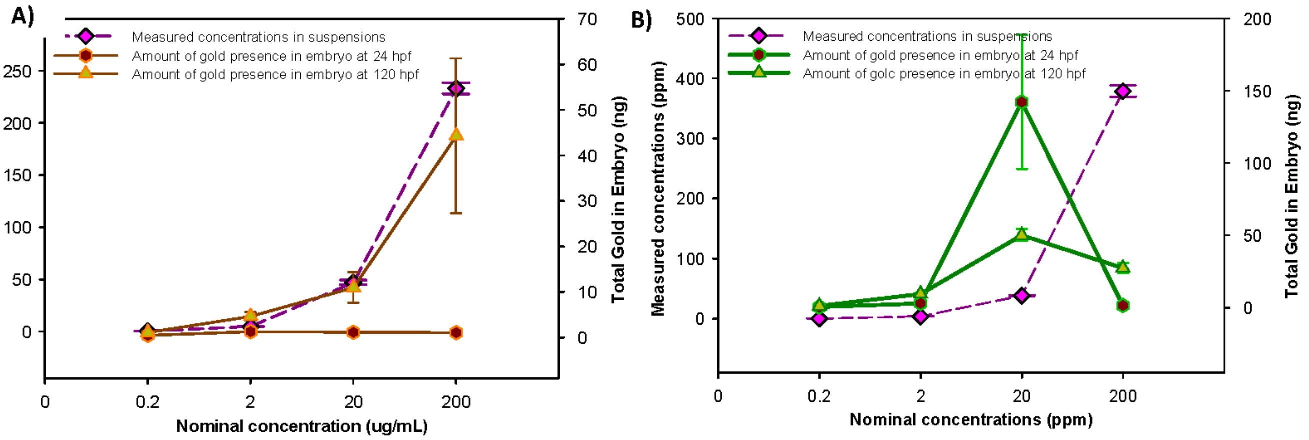

References

1. Liu, X.; Atwater, M.; Wang, J.; Huo, Q. Extinction coefficient of gold nanoparticles with different sizes and different capping ligands. *Coll. Surf. B Biointerfaces* **2007**, *58*, 3–7.

© 2014 by the authors; licensee MDPI, Basel, Switzerland. This article is an open access article distributed under the terms and conditions of the Creative Commons Attribution license (http://creativecommons.org/licenses/by/3.0/).
